# Lipophosphoglycan From Dermotropic New World *Leishmania* Upregulates Interleukin-32 and Proinflammatory Cytokines Through TLR4 and NOD2 Receptors

**DOI:** 10.3389/fcimb.2022.805720

**Published:** 2022-03-23

**Authors:** Murilo Barros Silveira, Rodrigo Saar Gomes, Marina Tiemi Shio, Jeronimo Nunes Rugani, Larissa Ferreira Paranaiba, Rodrigo Pedro Soares, Fátima Ribeiro-Dias

**Affiliations:** ^1^ Laboratório de Imunidade Natural (LIN), Instituto de Patologia Tropical e Saúde Pública, Universidade Federal de Goiás, Goiânia, Brazil; ^2^ Programa de Pós-graduação em Saúde Pública, Universidade Santo Amaro, São Paulo, Brazil; ^3^ Instituto René Rachou, Fundação Oswaldo Cruz, Belo Horizonte, Brazil

**Keywords:** IL-32, lipophosphoglycan, *Leishmania braziliensis*, *Leishmania amazonensis*, TLR4, NOD2

## Abstract

Interleukin-32 (IL-32) is produced during *Leishmania* infection, but the components of the parasite that induce its production are unknown. An important multivirulence factor of *Leishmania* spp. protozoa is the lipophosphoglycan (LPG), which plays a crucial role in the host-parasite interaction. Here, the ability of LPGs from two dermotropic *Leishmania* species to induce IL-32 production was evaluated in human peripheral blood mononuclear cells (PBMCs). Additionally, the potential receptors involved in this activation were assessed. PBMCs from healthy individuals were stimulated with LPGs from *L. amazonensis* (La) or *L. braziliensis* (Lb), live promastigotes of La or Lb and *E. coli* lipopolysaccharide (LPS, TLR4 agonist) as control. Blockers of TLR4 (*Bartonella quintana* LPS or monoclonal antibody) and Ponatinib (RIPK2 inhibitor, NOD2 pathway) were used to evaluate the receptors. ELISA was performed for IL-32 expression and cytokine (IL-1β and IL-6) production in cell lysates and in supernatants, respectively. Expression of TLR4 (2 h, 24 h) was assessed by flow cytometry. IL-32γ mRNA transcript was analyzed by qPCR. It was observed that LPG from *Leishmania*, like whole parasites, induced the production of IL-32, IL-1β and IL-6. Both LPGs induced the expression of *IL32*γ mRNA. The production of IL-32 was earlier detected (6 h) and positively associated with the production of IL-1β and IL-6. The induction of cytokines (IL-32, IL-1β and IL-6) was dependent on TLR4 and NOD2. The TLR4 was internalized after interaction with LPG. Therefore, our data suggest that LPGs from La and Lb are components of *Leishmania* able to upregulate IL-32 and other pro-inflammatory cytokines in a TLR4- and NOD2-dependent manner. In addition, LPG-induced IL-32 seems to be necessary for IL-1β and IL-6 production. To identify the parasite factors and host receptors involved in IL-32 induction is crucial to reveal potential targets for novel strategies to control leishmaniasis.

## Introduction

Leishmaniases are a group of neglected diseases, endemic especially in subtropical and tropical regions caused by *Leishmania* protozoan (Alvar et al., 2012). During the blood meal of insect vectors, *Leishmania* metacyclic promastigote forms are regurgitated on the host skin leading to the development of an innate immune response. Parasites are phagocytosed by neutrophils and mononuclear phagocytes including monocytes, macrophages, and dendritic cells (DCs) (Peters et al., 2008). Inside macrophages, promastigotes differentiate into amastigotes, proliferate, and infect new cells, triggering an inflammatory process (Gontijo and de Carvalho M de, 2013; Rogers et al., 2009).

In lesions of patients with American tegumentary leishmaniasis (ATL), there is a high expression of the pro-inflammatory cytokine interleukin (IL)-32γ (Galdino et al., 2014; Gomes et al., 2017). This cytokine is induced by *L. braziliensis* or *L. amazonensis* in human macrophages. It favors the control of the parasites through the production of nitric oxide (NO) and antimicrobial peptides (dos Santos et al., 2017). Recently, the functional consequences of three genetic variants of *IL32* modulating the expression of IL-32γ were reported. After exposure to *Leishmania*, they affected innate and adaptive cytokine production (Santos et al., 2020). In addition, treatment of monocyte-derived macrophages with recombinant IL-32γ and/or with IL-15 reduced *L. braziliensis* infection in a vitamin D-dependent manner (Silva LL de et al., 2020). Despite our knowledge about the effects of IL-32 on *Leishmania* infection, the parasite molecules that induce this cytokine have not been identified so far.

Lipophosphoglycan (LPG) is a major multivirulence factor expressed on the *Leishmania* promastigote surface. This molecule is essential during host-parasite interaction either in the invertebrate or vertebrate hosts (de Assis et al., 2012). Several functions are described for LPG including: binding to the insect’s midgut (Kamhawi et al., 2004), resistance to digestive enzymes (Borovsky and Schlein, 1987), resistance to complement (Brittingham and Mosser, 1996), modulation of phagosome maturation (da Silva Vieira et al., 2019), triggering of TLR2/TLR4 (de Veer et al., 2003; Ibraim et al., 2013), modulation of NO/cytokines and translocation of NF-kB (Paranaíba et al., 2015), induction of neutrophil extracellular traps (NETs) (Guimarães-Costa et al., 2009), induction of protein kinase R (PKR) (Vivarini et al., 2011), and induction of leukotriene B_4_ (LTB_4_) and prostaglandin E_2_ (PGE_2_) (Tavares et al., 2014; Lima et al., 2017). Several reports have focused on the immunomodulatory properties of LPGs from dermotropic/viscerotropic New World species of *Leishmania* including *L. amazonensis* (Nogueira et al., 2016), *L. braziliensis* (Vieira et al., 2019), *L. enriettii* (Paranaíba et al., 2015) and *L. infantum* (Ibraim et al., 2013; Cardoso et al., 2020). In general, they are mediated by TLR4 and in a lesser extent by TLR2. A distinguished feature of the LPGs from dermotropic *Leishmania* spp. and/or strains is the most pro-inflammatory activity of them compared to those from viscerotropic strains (Nogueira et al., 2016; Vieira et al., 2019; Cardoso et al., 2020). LPG also activates non-canonical NLRP3 inflammasome increasing IL-1β production by murine macrophages (de Carvalho et al., 2019). However, as most of these LPG effects were demonstrated in mice and IL-32 is not expressed in these animals, it is crucial to evaluate whether and through which receptors *Leishmania* glycoconjugates can modulate IL-32 production in human cells.

*Leishmania braziliensis* and *L. amazonensis* are the causative agents of ATL that lead to a spectrum of clinical outcomes ranging from single cutaneous lesions to severe forms like mucocutaneous or anergic diffuse leishmaniasis (Marques et al., 2017). Interspecies polymorphisms occur in the LPGs from those two species and are determinant for the immunopathological events during innate immune responses (Nogueira et al., 2016; Vieira et al., 2019). The LPG of *L. braziliensis* is devoid of side chains (Soaresa et al., 2005), whereas that of *L. amazonensis* possess galactoses and glucoses branching off the Gal(β1,4)Man(α1)-PO_4_ repeat unit motifs (Nogueira et al., 2017). Those biochemical polymorphisms resulted in differential murine macrophage stimulation through TLR2/TLR4 triggering different patterns of cytokine and NF-kB translocation (Ibraim et al., 2013; Nogueira et al., 2016; Vieira et al., 2019). These studies indicated that LPG interspecies polymorphisms could also affect IL-32 induction in human cells.

In fact, we have shown that induction of IL-32γ by *L. amazonensis* is dependent on NOD2 and TLR4 while by *L. braziliensis* is dependent on TLR4 but not on NOD2, in human peripheral blood mononuclear cells (PBMCs) (Santos et al., 2020). These results reinforced the need of evaluation of LPG interspecies polymorphisms in the IL-32γ production. As part of a broader study on functional properties of LPGs from dermotropic *L. amazonensis* or *L. braziliensis*, here we investigated their ability to induce IL-32 in human cells. The role of TLR4 and NOD2 as receptors for LPG-mediated effects was also investigated.

## Methods

### Ethics Statement

This study was approved by the Research Ethics Committee of Hospital das Clínicas/UFG, protocol CAAE: 44033514.0.0000.5078. Blood was collected from blood donors from Instituto Goiano de Oncologia e Hematologia (INGOH); individuals were over 18 years old and of both sexes. Before blood collection, patients read and signed the Informed Consent Form.

### Parasite’s Culture

*Leishmania* (*Leishmania*) *amazonensis* (IFLA/BR/67/PH8) and *Leishmania* (*Viannia*) *braziliensis* (MHOM/BR/2003/IMG) were cultured as promastigote forms in Grace’s insect medium (Gibco Life Technologies, USA) supplemented with 20% heat-inactivated fetal bovine serum (FBS, Gibco Life Technologies), 100 U/mL of penicillin/streptomycin (Sigma-Aldrich, USA) at 26°C. Stationary-phase parasites were obtained on the 6th day of growth and washed three times with phosphate-buffered saline (PBS; 1,000 g, 10 min, 10°C).

### Extraction and Purification of Lipophosphoglycans

The extraction and purification of LPGs were described in Nogueira et al. (2016). Briefly, late log-phase parasites were harvested and washed twice with PBS prior to LPG extraction with solvent E (H_2_O/ethanol/diethylether/pyridine/NH_4_OH; 15:15:5:1:0.017). For purification, the solvent and extract were dried under N_2_ evaporation, resuspended in 2 mL of 0.1 N acetic acid/0.1 M NaCl, and subjected to hydrophobic chromatography using a column of phenyl-Sepharose. The column was sequentially washed with 6 mL of 0.1N acetic acid/0.1 M NaCl, 1 mL of 0.1 N acetic acid and 1 mL of endotoxin free water. The LPG was eluted with 4 mL of solvent and then dried under N_2_ evaporation. LPG concentration was determined using the phenol-sulfuric method. All solutions were prepared in sterile LPS-free distilled water (Sanobiol, Campinas, Brazil).

### Obtaining Human Peripheral Blood Mononuclear Cells and Treatments

Blood (20 mL) was obtained by venipuncture, collected in vacuum tubes containing EDTA anticoagulant (BD, Brazil). Blood was diluted v/v in PBS-EDTA 0.01 mM pH 7.3, and layer on 1.077 density gradient (Ficoll, GE Healthcare, Switzerland). After centrifugation (1400 g, 30 minutes, 4°C), PBMCs were harvested and cells were washed twice with PBS-EDTA (600 g, 10 minutes, 4°C); subsequently, cells were resuspended in RPMI 1640 medium (Sigma-Aldrich), supplemented with 2 mM L-glutamine (Sigma-Aldrich), 11 mM sodium bicarbonate, 100 U/ml penicillin, 100 μg/ml streptomycin (Sigma-Aldrich) and 10% FBS (Gibco Life Technologies), named as complete RPMI medium. Cell viability was evaluated with 0.1% trypan blue in PBS, and cells were used only when there was ≥ 90% viability. Cells were quantified in a hematocytometer and distributed as 5 x 10^5^ PBMCs/well/200 µL into 96-well U-bottom plates (Corning-Costar, USA).

PBMCs were cultured in the presence of LPG from *L. amazonensis* (La) or *L. braziliensis* (Lb) at 10 μg/mL as previously reported (Nogueira et al., 2016; Vieira et al., 2019), live stationary phase promastigotes (Pro) of La or Lb (10 parasites:1 cell) or recombinant human IL-15 (rhIL-15; 100 ng/mL) (Silva LL de et al., 2020), at 37 °C, 5% CO_2_, for 24 h, in the presence of polymyxin B (10 μg/mL; Sigma-Aldrich; polymyxin B was used to rule out possible effects of contaminating LPS). Before infections or treatments, in some experiments, PBMCs were pretreated or not, for 1 h with LPS from *Bartonella quintana* (100 ng/mL) (Popa et al., 2007), neutralizing antibody anti-TLR4 (1 µg/mL; clone HTA125, ABCAM, catalog n. ab30667) and RIPK2 inhibitor (Ponatinib 100 nM; Selleckhem, Houston, USA), at 37°C/5% CO_2_. LPS from *Bartonella* and Ponatinib, alone or in combination, do not present cellular toxicity, as assessed by the MTT assay (Kumar et al., 2018) and shown in **Figure S1**.

### Measurement of Cytokines

Cells were lysed with PBS containing 0.1% Triton X-100 for intracellular IL-32. The amount of IL-32 was determined by immunoassay (ELISA) kit, following the manufacturer’s instructions (R&D Systems, USA). The detection limit of IL-32 was 31 pg/mL. The concentrations of IL-1β and IL-6 were determined in cell culture supernatants by ELISA kits, following the manufacturer’s instructions; IL-1β (BD Bioscience, DC, USA) and IL-6 (Biolegend, San Diego, CA, USA). The detection limit was 15.62 pg/mL for both cytokines.

### Detection of IL-32γ Isoform

RNA was isolated from PBMCs stored in Trizol as previously described (Heinhuis et al., 2016). cDNA was prepared using the iScript kit (Bio-Rad). Primer pairs for the *IL32γ* isoform were previously described (Galdino et al., 2014) and were produced by Sigma-aldrich. Diluted cDNA was used for qPCR using the Step One Plus qPCR system (Applied Biosystems, FosterCity, CA, USA) with SYBR Green Mastermix (Applied Biosystems). Fold change and relative expression were calculated with the 2^-ddCT method normalized against the house keeping gene GAPDH.

### Analysis of TLR4 Internalization by Flow Cytometry

PBMCs (5 x 10^5^/well) were cultured in the presence of LPG (10 μg/mL) of *L. amazonensis* or *L. braziliensis* for 2 h or 24 h, at 37°C/5% of CO_2_. Afterwards, in a first set of experiments, cells were fixed with 2% paraformaldehyde for 15 min and incubated with monoclonal antibodies to TLR4 (anti-human CD284, Alexa Fluor 488, Clone HTA 125, eBioscience, San Diego, CA, USA, catalog n. 53-9917-42) or isotype control (IgG2a K mouse Alexa Fluor 488, Clone eBM2a, eBioscience, catalog n. 53-4724-80), for 15 min, to analyze TLR4 expression on the cell membrane. Cells were washed twice with PBS and fixed in 1% paraformaldehyde. For analysis of total expression (cell membrane and intracellular) of TLR4, cells were permeabilized with 0.3% saponin in PBS, and treated with anti-TLR4 as described above. In a second set of experiments, 2h-treated cells were incubated with anti-TLR4 (AlexaFluor 488; for membrane TLR4) for 20 min, washed and fixed with 1% paraformaldehyde. Then, the cells were permeabilized with 0.3% saponin in PBS and incubated with unconjugated anti-TLR4 (Thermo Scientific, anti-human CD284, Clone 76B357.1, catalog n. MA5-16216), for 20 min, washed and incubated for additional 20 min with anti-mouse IgG (H+L)-Texas Red (Sigma-Aldrich, catalog n. SAB3701026; for intracellular TLR4),. After fixation with 1% paraformaldehyde, acquisition (10,000 events in the monocyte gate, FSC x SSC) was performed using an ACCURI C6 cytometer (BD Bioscience). For analysis, the FSC v.4 program (DNS, Los Angeles, CA, USA) was used. In the first set of experiments, in non-permeabilized cells, we consider TLR4 expressed on cell membrane while in permeabilized cells, we consider total TLR4 expression as it includes cell membrane and intracellular TLR4. Intracellular TLR4 expression was obtained by subtracting total expression from membrane expression of TLR4. Data are represented as percent inhibition (%) relative to the untreated control. In the second set of experiments,…the two fluorochromes were analyzed on the same cells.

### Statistical Analysis

Data are presented as individual values, quartiles, maximum and minimum values, and medians. The results were evaluated using the Wilcoxon test to compare two paired samples, and the Anova Kruskal-Wallis test followed by Dunn’s test to compare three or more samples. Results expressed as mean and Standard Error of the Mean (SEM) were analyzed by Two-way Anova followed by Bonferroni’s *post hoc* test. Spearman’s correlation test was also used. GraphPad Prism 5.0 Software Inc. (San Diego, CA, USA) was used for graphs and analyses. The level of significance established was p < 0.05.

## Results

### Lipophosphoglycans From *L. braziliensis* and *L. amazonensis* Induce IL-32 Production in Human Cells

**Figure 1:**
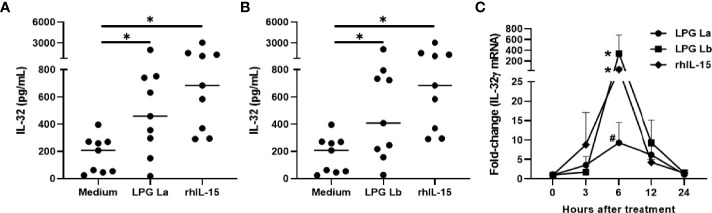
Lipophosphoglycans from *Leishmania* spp. induces IL-32 production and IL-32γ expression in human mononuclear cells. PBMCs (5 x 10^5^ cells/200 µL) were stimulated with LPG (10 µg/mL) of **(A)** *L. amazonensis* (La), **(B)** *L. braziliensis* (Lb) or **(A, B)** rhIL-15 (100 ng/mL). Cell lysates were obtained after 24 h of culture and IL-32 was measured by ELISA. **(C)** PBMCs were incubated for different periods with LPG (10 µg/mL) from *L. amazonensis* (La) or from *L. braziliensis* (Lb) or rhIL-15 (100 ng/mL). The IL-32γ mRNA transcripts were analyzed and quantified by qPCR, using the GAPDH gene as a reference gene. In **(A, B)**, the data represent the median and individual values (n = 9). *p < 0.05, in relation to the control (Anova Kruskal-Wallis followed by Dunn’s *post hoc* test). In **(C)** data represent mean and standard error of mean (n = 6). *p < 0.05 (vs. time 0); ^#^p = 0.06 (vs. time 0) (Two-way Anova followed by Bonferroni’s *post hoc* test).

PBMCs were stimulated with LPG from *L. amazonensis* or *L. braziliensis*, live promastigotes or rhIL-15 for 24 h. As expected, rhIL-15 induced IL-32; surprisingly, LPGs from *La* or *Lb* induced IL-32 production at similar levels of those induced by rhIL-15 (**Figures 1A, B**). In addition, the LPGs from *La* (**Figure S2A**) or *Lb* (**Figure S2B**) induced similar amounts of IL-32 as live promastigote forms did (MOI 10:1). No differences were detected between levels of IL-32 induced by LPGs from both species (**Figure S2C**).

IL-32γ is the most expressed isoform in *Leishmania* infection (Galdino et al., 2014; dos Santos et al., 2017), suggesting that LPG could induce IL-32γ as well in PBMCs. In fact, both LPGs or rhIL-15 were able to induce *IL32γ* mRNA. The time course of *IL-32γ* mRNA expression was similar for the three stimuli with peaking at 6 h of incubation (**Figure 1C**); however *L. amazonensis* LPG showed lower transcript levels at 6 h compared to *L. braziliensis* LPG and rhIL-15 (**Figure 1C**). These data suggest that both LPGs can induce the expression and production of IL-32γ in human cells.

### Lipophosphoglycans From *L. braziliensis* and *L. amazonensis* Induce IL-32, IL-6 and IL-1β Production in a TLR4-Dependent Manner

**Figure 2:**
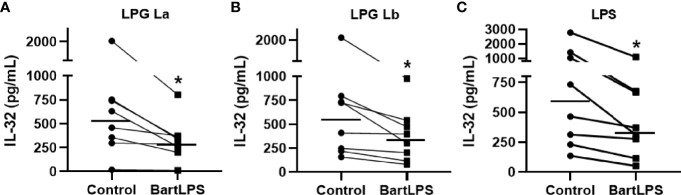
Induction of IL-32 by lipophosphoglycans from *Leishmania* spp. occurs in a TLR4-dependent manner. Peripheral blood mononuclear cells (5 x 10^5^ cells/200 µL) were treated or not with LPS from *Bartonella quintana* (BartLPS, 100 ng/ml) for 1 h. After the incubation time, cells were stimulated with LPG (10 µg/ml) of **(A)** *L. amazonensis* (La) or from **(B)** *L. braziliensis* (Lb) or **(C)** LPS from *E. coli* (100 ng/mL), for an additional 24 h. Cell lysates were obtained for IL-32 evaluation by ELISA. IL-32 production was induced by LPG of *L. amazonensis* **(A)**, *L. braziliensis* **(B)** and *E. coli* LPS **(C)**. Data represent individual measurements and median values (n = 8). *p < 0.05 (vs. control) by Wilcoxon test.

*L. mexicana*, *L. braziliensis* and *L. amazonensis* LPGs induce the production of pro-inflammatory cytokines in human and murine macrophages *via* TLR4 (Rojas-Bernabé et al., 2014; Nogueira et al., 2016; Vieira et al., 2019). We therefore investigated whether the induction of IL-32 by LPGs from *L. amazonensis* and *L. braziliensis* occurs in a TLR4-dependent manner in human cells. Indeed, blocking of TLR4 with LPS from *Bartonella quintana* (BartLPS), a natural TLR4 antagonist (Popa et al., 2007), resulted in a reduction of IL-32 production induced by *L. amazonensis* LPG (**Figure 2A**) or *L. braziliensis* LPG (**Figure 2B**) as well as by LPS from *E. coli* (**Figure 2C**). These results were confirmed by blocking of TLR4 with neutralizing antibodies (**Figure S3A**). Thus, suggesting that the induction of IL-32 by LPGs from La or Lb is dependent on TLR4.

**Figure 3:**
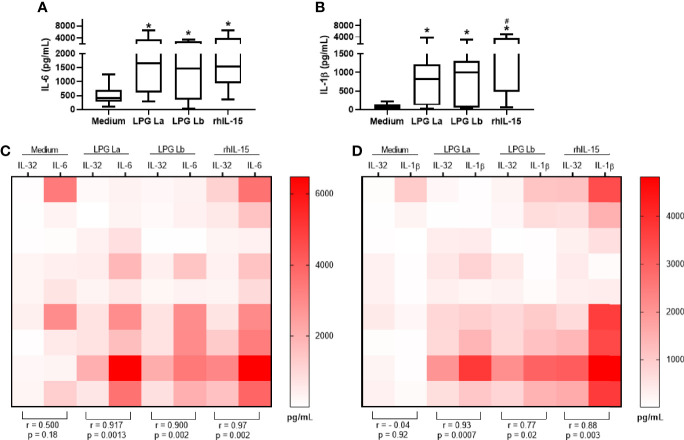
IL-32 production is positively correlated with IL-1β and IL-6 production in human cells stimulated with lipophosphoglycans from *Leishmania* spp. PBMCs (5 x 10^5^ cells/200 µl) were stimulated with LPG (10 µg/mL) from *L. amazonensis* (La) or from *L. braziliensis* (Lb) or with rhIL-15 (100 ng/mL). Supernatants were collected after 24 h of culture for IL-1β **(A)** and IL-6 **(B)** cytokine evaluation. In **(C, D)**, heat maps and correlations between IL-32 (measured in cell lysates) in comparison to IL-6 and IL-1β levels, respectively. Data represent medians, quartiles and minimum values and maximum (n = 9 donors). In **(A, B)**, *p < 0.05 (vs. Medium); ^#^p < 0.05 (comparison between LPG of La and Lb) by Anova Kruskal-Wallis followed by Dunn’s *post hoc* test. In **(C, D)**, r (Spearman’s correlation test) and p values (n = 9 donors) are shown.

IL-32 induces pro-inflammatory cytokines such as IL-6 and IL-1β in human cells (Netea et al., 2005; Netea et al., 2008). Thus, we decided to investigate whether the LPGs of La and Lb induce the production of proinflammatory cytokines and whether this production is associated with the production of IL-32. We observed that both LPGs induced the production of IL-1β (**Figure 3A**) and IL-6 (**Figure 3B**) in a TLR4-dependent manner (**Figure S3B, C**, and **Figure S4**). After cell activation with LPGs, positive correlations were observed between the amounts of IL-32 and IL-6 (**Figure 3C**) or IL-1β (**Figure 3D**). The strongest associations occurred between *L amazonensis* LPG-induced IL-32 vs. IL-1β concentrations (r = 0.93) or rhIL-15-induced IL-32 vs. IL-6 (r = 0.97) (**Figures 3C, D**). Correlations between IL-32 vs. IL-6 concentrations, after stimulation of cells with LPG from the two *Leishmania* spp. or rhIL-15 were similar (**Figure 3C**). The data suggested that IL-32 was associated with LPG-induced pro-inflammatory cytokine production. In fact, time course of the production of pro-inflammatory cytokines, IL-6 and IL-1β, showed that these cytokines are produced later after the production of IL-32 by PBMCs stimulated with both LPGs (**Figure S5**).

### Lipophosphoglycans From *L. braziliensis* and *L. amazonensis* Induce TLR4 Internalization in Human Cells

**Figure 4:**
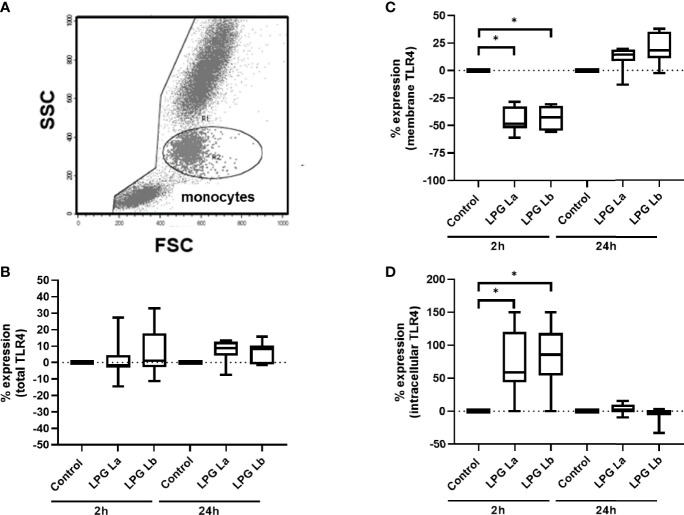
Lipophosphoglycans from *Leishmania* spp. induces TLR4 internalization. PBMCs (5 x 10^5^ cells/200 μL) were incubated in the presence of LPG (10 μg/mL) from *L. amazonensis* (La) or *L. braziliensis* (Lb) for 2 h or 24 h of incubation. The cells were fixed and TLR4 expression was assessed by flow cytometry, defining the monocyte population by FSC-H x SSC-H. Results are shown as percent of TLR4 immunofluorescence intensity (MFI) in LPG-treated cells relative to MFI of control cells incubated in the absence of LPG. **(A)** Gate for the population of interest. **(B)** Total TLR4 expression (membrane + intracellular) after 2 h or 24 h **(C)** TLR4 membrane expression after 2 h or 24 h incubation. **(D)** Intracellular TLR4 expression (total minus membrane) after 2 h or 24 h of incubation. Data represent medians, interquartiles, and minimum and maximum values ​​(n = 4 donors, evaluated in two independent experiments). *p < 0.05 (vs. control), by Anova Kruskal-Wallis followed by Dunn’s *post hoc* test.

We have previously shown that after interaction between *L. braziliensis* amastigotes and TLR4 there is internalization of this receptor (Galdino et al., 2016). Here, TLR4 expression was analyzed in the monocyte population, separated according to size (FSC-H) and cell complexity (SSC-H) (**Figure 4A**). In the first set of experiments, we observed that the expression of total TLR4 (membrane + intracellular) was not altered by incubating the cells with both LPGs for 2 h or 24 h (**Figure 4B**). However, TLR4 expression on monocyte membrane significantly decreased after 2 h of incubation with both LPGs (**Figure 4C**) in parallel with an increase in intracellular TLR4 (**Figure 4D**). After 24 h of incubation with LPGs, membrane and intracellular TLR4 expression levels were similar to the initial values (**Figures 4B, D**). In a second set of experiments, when we evaluated the expression of TLR4 by using antibodies with different fluorochromes, for membrane and intracellular (**Figure S6A**), we confirmed the internalization of TLR4 after treatment of cells with LPG from both *Leishmania* spp. (**Figure S6B**). These data suggest that both LPGs interact and are internalized with TLR4.

### Lipophosphoglycans From *L. braziliensis* and *L. amazonensis* Induce NOD2-Dependent Production of IL-32 and Pro-Inflammatory Cytokines in Human Mononuclear Cells

**Figure 5:**
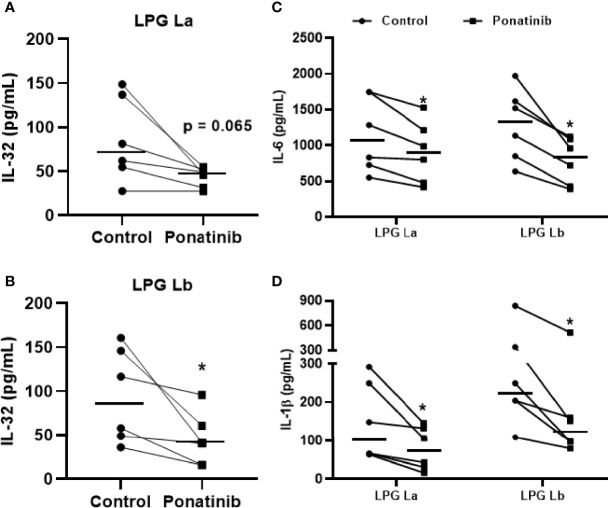
Induction of IL-32 by lipophosphoglycans from *Leishmania* spp. is dependent on NOD2. PBMCs (5 x 10^5^ cells/200 μL) were treated or not with Ponatinib (100 nM) for 1 h and stimulated with LPG (10 μg/mL) from *L. amazonensis* (La) or from *L. braziliensis* (Lb) for 24 h. Cell lysates were obtained for IL-32 evaluation by ELISA, and IL-32 production is shown after exposure to LPG from *L. amazonensis* **(A)** or *L. braziliensis* **(B)**. Supernatants were collected for IL-6 **(C)** or IL-1β **(D)** evaluation by ELISA. Data represent individual and median values (n = 6 donors, evaluated in three independent experiments). *p < 0.05 (vs. control) by Wilcoxon test.

Dos Santos et al. (2017) (Dos Santos et al., 2017) demonstrated that the NOD2 receptor in human monocytes/macrophages is crucial for immune responses against *Leishmania* and intracellular control of the parasites. Next, we evaluated whether the NOD2 signaling pathway would also be important for the production of IL-32 and other pro-inflammatory cytokines using Ponatinib. This compound is a potent inhibitor of RIPK2 phosphorylation, an essential kinase for the NOD2 signaling cascade and production of inflammatory cytokines (Dos Santos et al., 2017). Blocking the RIPK2 phosphorylation pathway caused a reduction in IL-32 production induced by La LPG (**Figure 5A**) and Lb LPG (**Figure 5B**). Inhibition of NOD2 pathway by Ponatinib also reduced the production of IL-6 (**Figure 5C**) and IL-1β (**Figure 5D**) by LPG-stimulated human cells. These data suggest that both LPGs induce the production of IL-32, IL-6 and IL-1β *via* NOD2 pathway.

**Figure 6:**
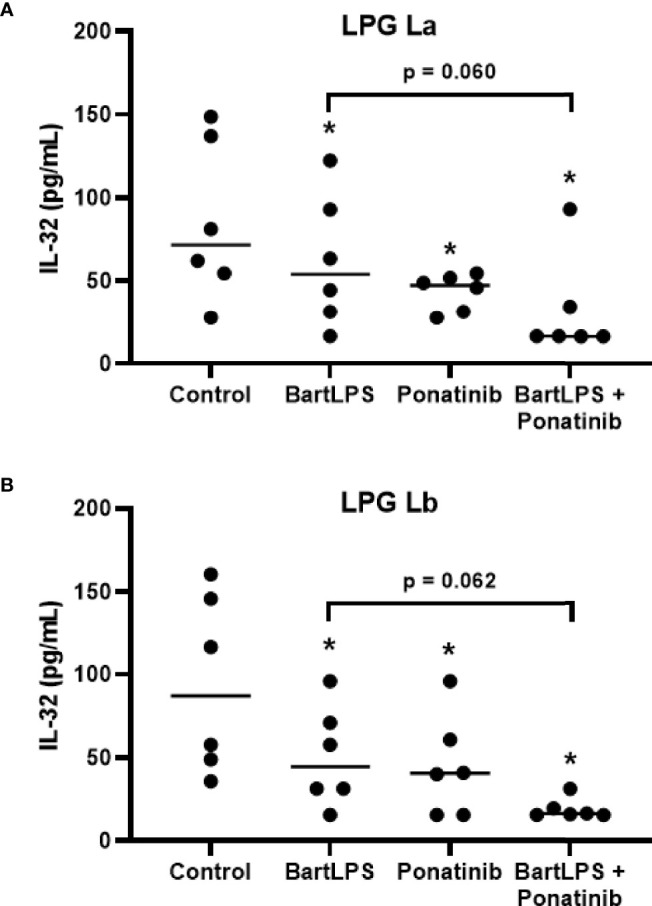
Induction of IL-32 by lipophosphoglycans from *Leishmania* spp. is dependent on TLR4 and NOD2. PBMCs (5 x 10^5^ cells/200 μL) were treated or not with LPS from *Bartonella quintana* (100 ng/mL) and/or Ponatinib (100 nM) for 1 h. Then, cells were incubated with LPG (10 μg/mL) from *L. amazonensis* (La; **A**) or *L. braziliensis* (Lb; **B**) for 24 h. Cell lysates were obtained for the IL-32 evaluation by ELISA. Data represent individual and median values (n = 6 donors, evaluated in three independent experiments). *p < 0.05 (vs. control) by Anova Kruskal-Wallis followed by Dunn’s *post hoc* test.

Simultaneous antagonism of the TLR4 and inhibition of NOD2 pathway reduced IL-32 production by La LPG (**Figure 6A**) and Lb LPG (**Figure 6B**), indicating that both receptors are contributing for LPG effects.

## Discussion

The results of this study showed that LPGs from *L. amazonensis* and *L. braziliensis* induced IL-32 production in human cells, similarly to rhIL-15, a potent IL-32 inducer (Montoya et al., 2014). The amounts of IL-32 produced after stimulation with the LPGs from both species of *Leishmania* were similar to those induced by live promastigote forms. Whereas the production of IL-32 was not significantly different when comparing the two species, less amount of mRNA IL-32γ was expressed after exposure to *L. amazonensis* LPG than to *L. braziliensis* LPG. Dos Santos et al. (2017) showed that promastigote forms of *L. amazonensis* induced higher expression of mRNA *IL32γ* and production of IL-32 than metacyclic forms of *L. braziliensis* in human macrophages differentiated from the monocytic lineage THP-1 (dos Santos et al., 2017). It is noteworthy, therefore, that differences between a differentiated cell line (THP-1) and primary monocytes exist (Chanput et al., 2012) and may explain these differences. Monocytes, the main source of IL-32 in PBMCs (Netea et al., 2006) and THP-1 macrophages can present differential transcriptional and post-transcriptional mechanisms to control the production of IL-32 induced by different *Leishmania* species. In PBMCs, the data obtained in the present study suggest that the LPG from *L. braziliensis* induces more efficient transcriptional pathways than *L. amazonensis* LPG but may be tighter control of translation. Alternatively, the expression/production of other IL-32 isoforms could explain these discrepancies.

The structures of *L. amazonensis* and *L. braziliensis* LPGs are polymorphic in composition and displayed different functional properties in murine macrophages (Soaresa et al., 2005; Nogueira et al., 2016; Vieira et al., 2019). Although they are both TLR4 agonists and induced NO, IL-6, and TNF-α, only the LPG of *L. braziliensis* was able to induce IL-12 and IL-1β (Ibraim et al., 2013; Vieira et al., 2019). Data from the present study suggest that despite their polymorphisms in the sugar motifs, differences in sugar composition of *L. amazonensis* and *L. braziliensis* did not significantly influence the amounts of IL-32 induced by both *Leishmania* species in PBMCs. Consistent with those observations, LPGs from two *L. amazonensis* strains (PH8 and Josefa) did not differ in their immunomodulatory properties in murine macrophages (Nogueira et al., 2016). However, this appears not to be the case for *L. braziliensis* LPGs where a larger number of strains from different clinical forms was employed. Thus, for both strains employed in this study, the lipid component may have been determinant for the induction of IL-32, a similar feature to LPS, which uses the lipid A component to stimulate TLR4 pathway and induces IL-32.

TLRs are a well-characterized class of pattern recognition receptors (PRRs) used by phagocytes to recognize PAMPs of infectious agents as *Leishmania* (Ozinsky et al., 2000). Activation of TLRs by parasite molecules triggers the activation of the transcription factor NF-κB and the MAPK pathway to induce the expression of pro-inflammatory cytokine genes that are essential to control parasite replication (T et al., 2008). TLR4 is reported to be an important receptor for the development of the inflammatory response during leishmaniasis (Mukherjee et al., 2016). In the present study, it was observed that blocking of TLR4 with BartLPS, a natural TLR4 antagonist (Popa et al., 2007), as well as with monoclonal antibodies caused a reduction in LPG-induced IL-32 production by both species of *Leishmania*. These data suggest that TLR4 recognizes LPG and is at least partially responsible for the IL-32 induction. *L. braziliensis* LPG was able to differentially modulate macrophage functions through activation of TLR2 and TLR4 (de Assis et al., 2012; Ibraim et al., 2013). Regarding the *L. mexicana* complex, of which *L. amazonensis* is part, a study demonstrated the inflammatory role of LPG *via* the binding of TLR2 and TLR4 receptors (Rojas-Bernabé et al., 2014). Furthermore, infection of human cells with *L. braziliensis* increased the production of TNFα and IL-10 in a TLR4-dependent manner (Galdino et al., 2016), reinforcing the role of TLR4 as an important receptor in *Leishmania* infections. The data from the present study strengthen those from Dos Santos et al. (2020) where it was shown that TLR4 is an important receptor for IL-32 induction by lysates of *L. amazonensis* and *L. braziliensis* promastigote forms (Santos et al., 2020). Therefore, data from the present study suggest that the LPGs from *L. amazonensis* and *L. braziliensis* are PAMPs that induce IL-32 in human mononuclear cells in a TLR4-dependent manner.

As TLR4 activation leads to inflammation and it is known that IL-32 can induce pro-inflammatory cytokines (Netea et al., 2005; Netea et al., 2008; dos Santos et al., 2017), and that inflammatory cytokines induce IL-32 (Kim et al., 2005; Kang et al., 2014), we investigated the interactions between these cytokines after cell activation with LPGs. We observed that LPGs from both *Leishmania* spp. can induce the production of IL-6 and IL-1β, and a positive correlation was detected between the levels of IL-32 and those of both IL-6 and IL-1β. Moreover, blocking of TLR4 decreased the production of IL-6 and IL-1β after stimulation of PBMCs with LPGs. The time course of cytokine production after LPG treatment suggest that LPG induces IL-32, thereby inducing IL-1β and IL-6 in a TLR4-dependent manner. This suggestion is corroborated by our findings in a functional genomics study, which detected *IL32* variants influencing cytokine (IL-1β, TNF-α, IL-6) production upon exposure to lysates of both *Leishmania* species (Santos et al., 2020). Our data confirm the capacity of LPG to induce pro-inflammatory cytokines in a TLR4-dependent manner (Rojas-Bernabé et al., 2014; Nogueira et al., 2016; Vieira et al., 2019) and, in addition, show that this pathway is responsible for IL-32 induction, which is associated with IL-6 and IL-1β production. In fact, *L. mexicana*, *L. braziliensis* and *L. amazonensis* LPGs induce production of pro-inflammatory cytokines *via* TLR4, both in human and murine macrophages (Rojas-Bernabé et al., 2014; Nogueira et al., 2016; Vieira et al., 2019). However, it is important to mention that despite their abilities to induce NO and cytokine production in murine macrophages, only the LPG from *L. braziliensis* (strain M2903) was able to translocate NF-KB by CHO cells *via* TLR2 (Ibraim et al., 2013). A distinguished feature of the LPGs of *L. amazonensis* (PH8 and Josefa strains) is that they are very pro-inflammatory but unable to translocate NF-kB in CHO cells *via* TLR2/TLR4 (Nogueira et al., 2016). This paradoxical effect does not seem to be present in murine macrophages or human PBMCs evaluated here, highlighting possible differences in the cell models employed in different studies.

It is reported that TLR4 can be internalized and participate in endosome trafficking, thereby inducing inflammatory cytokines (Galdino et al., 2016). In the present study, it was observed that after treatment of PBMCs with LPG, the expression of total TLR4 (membrane and intracellular) in monocytes was not altered after 2 h or 24 h. However, the TLR4 expression on cell membrane significantly decreased after 2 h of incubation with LPGs from both *Leishmania* spp. in parallel to an increase of the intracellular TLR4. After 24 h of incubation with LPG, membrane and intracellular TLR4 expression levels were similar, indicating recycling or TLR4 expression. In lung cells activated with LPS *via* the TLR4 receptor, it has been shown that the responsiveness to LPS is dependent on the levels of TLR4 present on the cell surface membrane, which is determined by the amount of TLR4 that travels from the Golgi complex to the plasma membrane and the amount of TLR4 internalized (McGettrick and O'Neill, 2010). For LPG, as a TLR4 agonist, the same mechanisms can explain the results here; after exposure to LPG, TLR4 has been endocytosed and after a period of 24 h it was replenished on the cell surface. The regulatory pathways of TLR4-induced signaling by endocytosis and the factors that restrict these processes are just beginning to be elucidated. Zanoni et al. (2011) described a LPS-induced endocytic process that is dependent on the CD14 co-receptor, but independent of TLR4 signaling pathways (Zanoni et al., 2011). In that study, it was shown that the response depends on the cell type exposed to LPS and the co-expression of TLR4 and CD14 (Zanoni et al., 2011). Galdino et al. (2016) demonstrated that after exposure of PBMCs to amastigote forms of *L. braziliensis*, plasma membrane TLR4 molecules decreased on monocyte surface whereas increased intracellularly, suggesting their endocytosis together with the parasite (Galdino et al., 2016). This internalization was necessary for the production of TNFα and IL-10. As it is known that *Leishmania* amastigote forms do not present LPG on their surface, data suggested that other parasite molecules can interact with TLR4 in order to induce cytokine production as well (Galdino et al., 2016).

We have shown that NOD2 is receptor relevant for the control of *Leishmania* spp (Dos Santos et al., 2017). In addition, we reported that LPG was shown to activate NOD-like receptor NLRP3 in a non-canonical pathway (de Carvalho et al., 2019). Thus, in the present study we have inhibited the RIPK2 phosphorylation pathway, a critical kinase for the NOD2 signaling cascade and production of inflammatory cytokines (Park et al., 2007). Inhibition of the RIPK2 phosphorylation pathway caused a reduction in the IL-32 production induced by *L. amazonensis* LPG or *L. braziliensis* LPG, which in turn reduced the production of IL-1β and IL-6 in human cells. We have previously demonstrated that the NOD2 receptor in human macrophages is crucial for immune responses and intracellular control of infections caused by the same *Leishmania* species studied here. In individuals with mutations in the *NOD2* gene, the production of cytokines such as TNFα, IL-1β, IL-6, IL-8, IFNγ was significantly decreased in PBMCs after exposure to lysates or promastigote forms of *L. amazonensis* and *L. braziliensis* (Dos Santos et al., 2017). Additionally, in a study on visceral leishmaniasis, NOD2-RIPK2 activation contributed to the induction of a potent Th1 but inhibited the Th17 response, due to modulation of cytokine produced by DCs (Nascimento et al., 2016). How LPG accesses the cytoplasm to activate NOD2 remains unclear, but it could be possible that LPG escapes the endosome, after endocytosis with TLR4, and gains the cytosol, thereby activating NOD2. Production of IL-1β has been shown to be dependent on NLRP3 after LPG stimulation (de Carvalho et al., 2019). As the data from the present study suggested a synergism between TLR4 and NOD2 to induce IL-32 after exposure to LPG, it can be speculated that these receptors work together to induce IL-32 and IL-1β during *Leishmania* spp. infection.

**Figure 7:**
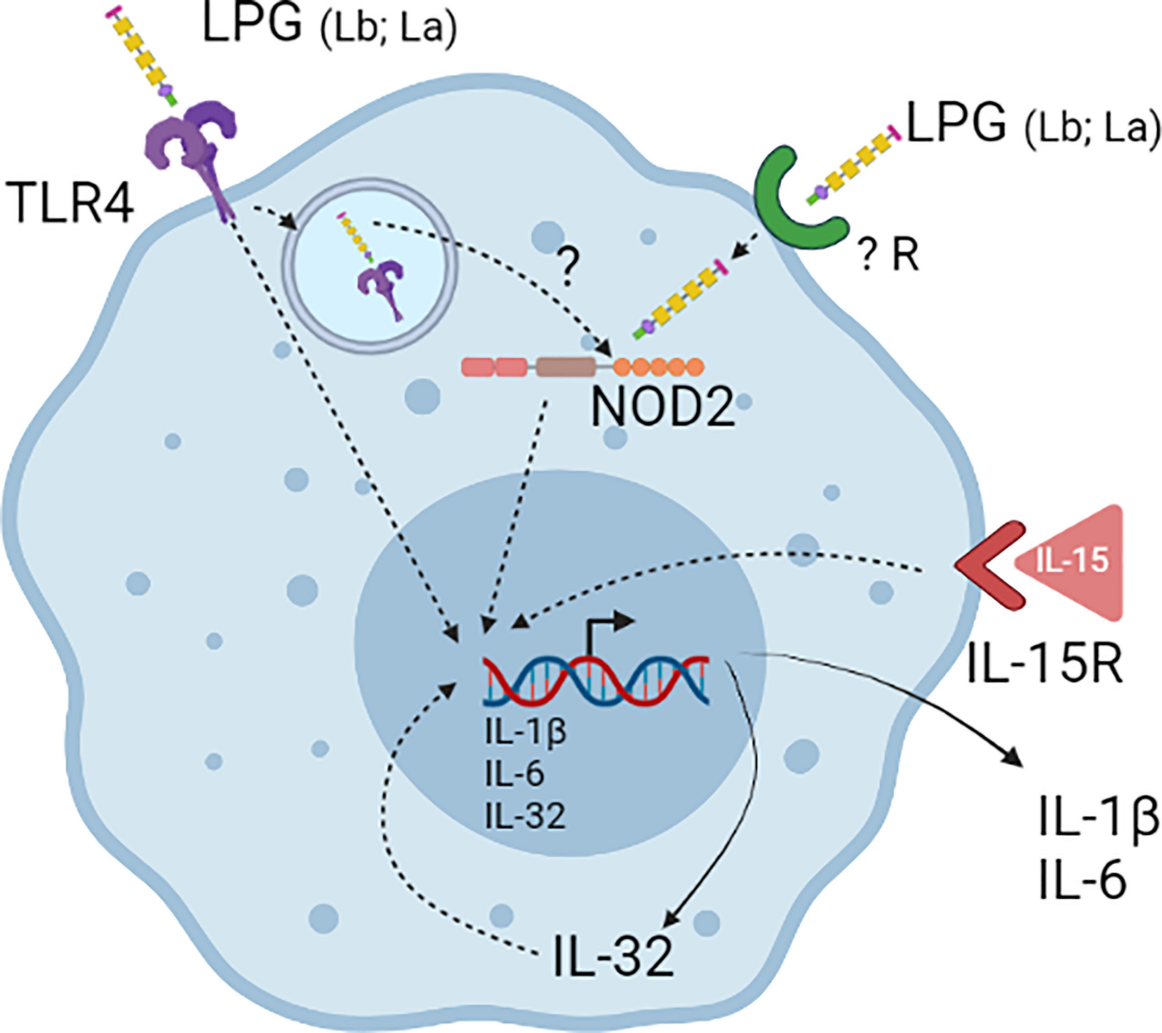
Lipophosphoglycans (LPGs) from *L. amazonensis* and *L. braziliensis* are molecules responsible for inducing IL-32 production. LPGs induce IL-32 in a TLR4- and NOD2-dependent manner in human PBMCs. After LPG endocytosis by TLR4 it can escape the endosomes and activate NOD2. IL-32 can increase the production of pro-inflammatory cytokines such as IL-6 and IL-1β induced by LPGs.

Most of the studies on functional properties of LPGs have been focused on murine macrophages and the studies with human cells are still scarce. A previous study with human neutrophils has shown the ability of *L. amazonensis* LPG to induce the production of LTB_4_ (Tavares et al., 2014). The present study also confirms the functional ability of LPGs from *L. amazonensis* and *L. braziliensis* as important PAMPs inducers of IL-32, IL-1β, and IL-6 production in human PBMCs. The induction of the cytokines is dependent on TLR4 and NOD2 receptors. In addition, data suggest that LPG-induced IL-32 is at least partially responsible for the IL-6 and IL-1β production induced by the LPGs. A landscape of the results is summarized in **Figure 7**. To identify the *Leishmania* PAMPs responsible for IL-32 induction is crucial to control the effects of this cytokine in leishmaniases and to the development of therapeutic and/or vaccine strategies for this important neglected disease.

## Data Availability Statement

The original contributions presented in the study are included in the article/**Supplementary Material**. Further inquiries can be directed to the corresponding author.

## Ethics Statement

This study was approved by the Research Ethics Committee of Hospital das Clínicas/UFG, protocol CAAE: 44033514.0.0000.5078. Blood was collected from blood donors from Instituto Goiano de Oncologia e Hematologia (INGOH); individuals were over 18 years old and of both sexes. Before blood collection, patients read and signed the Informed Consent Form. The patients/participants provided their written informed consent to participate in this study.

## Author Contributions

MBS, RG, MTS, JR, LP, RS, and FR-D participated in planning and/or performing the experiments. RG, RS, and FR-D conceived, planned the experiments, analyzed the results, and wrote the manuscript. All authors approved the submitted version.

## Funding

This work was supported by CAPES (Finance Code 001) and CNPq/FAPEG (grant n. 465771/2014-9 - INCT/HPI - National Institute of Science and Technology for the strategies in host-pathogen interaction), Brazil. FR-D and RS are researcher fellows of CNPq. RS is supported by CNPq (302972/2019-6), FAPEMIG (PPM-XII-00202-18 ) and FAPESP (2021/01243-0).

## Conflict of Interest

The authors declare that the research was conducted in the absence of any commercial or financial relationships that could be construed as a potential conflict of interest.

## Publisher’s Note

All claims expressed in this article are solely those of the authors and do not necessarily represent those of their affiliated organizations, or those of the publisher, the editors and the reviewers. Any product that may be evaluated in this article, or claim that may be made by its manufacturer, is not guaranteed or endorsed by the publisher.
