# Supplementary material for: Lipophosphoglycan From Dermotropic New World Leishmania Upregulates Interleukin-32 and Proinflammatory Cytokines Through TLR4 and NOD2 Receptors
Source: Front Cell Infect Microbiol. 2022 Mar 23;12:805720. doi: 10.3389/fcimb.2022.805720 (PMC8983857; doi:10.3389/fcimb.2022.805720)
Supplement: Supplementary file 1 [file DataSheet_1.docx]

**Supplementary Data**

**Figure S1. Cell viability in PBMCs treated with BartLPS and Ponatinib.** PBMCs (5 x 10^5^ cells/200 μL) were treated or not with LPS from *Bartonella quintana* (BartLPS, 100 ng/mL) and/or Ponatinib (100 nM) for 24 h. Cell viability was assessed by the MTT assay and compared with the untreated control (RPMI). Data represent individual values and medians from 4 donors, evaluated in two independent experiments.

**Figure S2. LPG-induced IL-32 production.** PBMCs (5 x 10^5^ cells/200 µL) were stimulated with LPG (10 µg/mL) of *L. amazonensis* (La), *L. braziliensis* (Lb) or stationary phase promastigotes (Pro) of both species (MOI 10:1). Cell lysates were obtained after 24 h of culture and IL-32 was measured by ELISA. IL-32 production induced by LPG or promastigote infection (Pro) of *L. amazonensis* (A), *L. braziliensis* (B) or only LPGs (C). Data represent the median and individual values (n = 9). *p ˂ 0.05, in relation to the control (Anova Kruskal-Wallis/Dunn’s post hoc test).

**Figure S3. Production of IL-32, IL-1β and IL-6 in human mononuclear cells stimulated with lipophosphoglycan from *Leishmania* spp. is TLR4 dependent.** PBMCs (5 x 10^5^ cells/200 μL) were treated or not with LPS from *Bartonella quintana* (BartLPS, 100 ng/mL) or anti-TLR4 neutralizing antibody (1 µg/mL) for 1 h and stimulated with LPG (10 μg/mL) from *L. amazonensis* (La) or *L. braziliensis* (Lb) or with LPS from *E. coli* (100 ng/mL) for an additional 24 h. Cell lysate (for IL-32) or supernatants were collected for cytokine evaluation by ELISA. (A) IL-32 production. (B) IL-6 production. (C) IL-1β production. Data represent individual values and medians from 4 donors, evaluated in two independent experiments. *p ˂ 0.05 Control vs BartLPS or anti-TLR4, in each treatment, by Anova Kruskal-Wallis/Dunn’s post hoc test.

**Figure S4. Production of IL-1β and IL-6 in human mononuclear cells stimulated with lipophosphoglycan from *Leishmania* spp. is TLR4 dependent.** PBMCs (5 x 10^5^ cells/200 μL) were treated or not with LPS from *Bartonella quintana* (BartLPS, 100 ng/mL) for 1 h and stimulated with LPG (10 μg/mL) from *L. amazonensis* (La) or *L. braziliensis* (Lb) or with LPS from *E. coli* (100 ng/mL) for an additional 24 h. Supernatants were collected for cytokine evaluation by ELISA. (A) IL-6 production. (B) IL1β production. Data represent individual values and medians from 6 donors, evaluated in two independent experiments. *p ˂ 0.05 Control vs BartLPS, by Wilcoxon test.

**Figure S5. Production of IL-6 and IL-1β is dependent on the production of IL-32 in human mononuclear cells stimulated by lipophosphoglycan from *Leishmania* spp**. PBMCs (5 x 10^5^ cells/200 μL) were incubated in the absence (medium) or presence of LPG (10 μg/mL) from *L. amazonensis* (Lb) or *L. braziliensis* (La), for 3 h to 24 h. The supernatants were collected for evaluation of pro-inflammatory cytokines and cell lysates were obtained for the evaluation of IL-32 by ELISA. Data show time-course of IL-32 (A), IL-6 (B) and IL-1β (C) production. Data represent the mean and standard deviation of 6 donors, in two independent experiments. *p ˂ 0.05, LPG La and LPG Lb vs. Medium (two-way Anova/Bonferroni).


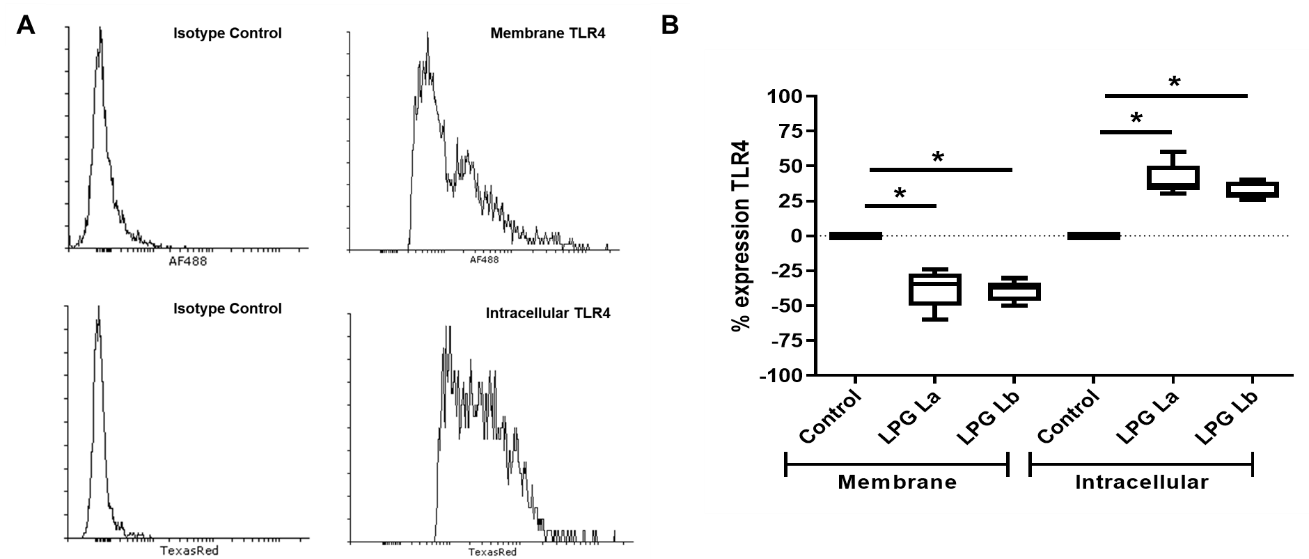


**Figure S6. Lipophosphoglycan from *Leishmania* spp. induces TLR4 internalization.** PBMCs (5 x 10^5^ cells/200 μL) were incubated in the presence of LPG (10 μg/mL) from *L. amazonensis* (La) or *L. braziliensis* (Lb) for 2 h. Cells were incubated with anti-TLR4 (AlexaFluor 488; membrane TLR4) for 20 min, washed and fixed with 1% paraformaldehyde. Then the cells were permeabilized with 0.3% saponin in PBS and incubated with unconjugated anti-TLR4 and anti-mouse IgG (H+L)-Texas Red (Texas Red; intracellular TLR4). (A) TLR4 expression was assessed by flow cytometry, defining the monocyte population by FSC-H x SSC-H. (B) Results are shown as percent of TLR4 immunofluorescence intensity (MFI) in LPG-treated cells relative to MFI of control cells incubated in the absence of LPG. (Data represent medians, interquartiles, and minimum and maximum values (n = 4 donors, evaluated in two independent experiments). *p < 0.05 vs. control, by Anova Kruskal-Wallis followed by Dunn’s post hoc test.
